# The Representation of White Matter in the Central Nervous System

**DOI:** 10.3389/fnana.2018.00102

**Published:** 2018-12-13

**Authors:** Robert Baud, Pierre Sprumont, Hans J. ten Donkelaar

**Affiliations:** ^1^Anatomy, Section of Medicine, Faculty of Science and Medicine, University of Fribourg, Fribourg, Switzerland; ^2^SIB Data mining, Swiss Institute for Bioinformatics, Geneva, Switzerland; ^3^Department of Neurology, Radboud University Medical Center and Donders Center for Medical Neuroscience, Nijmegen, Netherlands

**Keywords:** neuroanatomy, terminology, white matter, ontology, knowledge representation

## Abstract

The white matter of the central nervous system (CNS) is difficult to represent in anatomy because it is located predominantly “between” other anatomical entities. In a classic presentation, like a cross section of a brain segment, white matter is present and can be labeled adequately. Several appearances of the same entity are feasible on successive cross section views. The problem is the absence of a global view on long tracts, and more generally, the lack of a comprehensive classification of white matter pathways. Following the recent revision of the *Terminologia Anatomica* (TA, 1998), in particular the chapter on the nervous system, resulting in the *Terminologia Neuroanatomica* (TNA, 2017), the authors have developed a new schema for the representation of white matter. In this approach, white matter is directly attached to the CNS, and no longer considered as part of the brain segments. Such a move does not affect the content but redistributes the anatomical entities in a more natural fashion. This paper gives an overall description of this new schema of representation and emphasizes its benefits. The new classification of white matter tracts is developed, selecting the origin as the primary criterion and the type of tract as the secondary criterion.

## Introduction

At large, white matter coordinates communication between regions of the brain and the spinal cord. It is described by several nouns such as *tract, funiculus, fasciculus, commissure, lemniscus, fibers, decussation*, and *stria*. For convenience, in this article, they will be referred to as *tract* or alternatively as *pathway*. However, the above-mentioned specific names will continue to be used in the terminology, because they eventually bring some additional information to the named entity. There is a permanent discussion when building a terminology: how much a term should contribute to the definition of its referred entity. Short terms or longer versatile terms? Both have their advantages.

Any tract has an origin located in a cortical region or in a nucleus (or a group of nuclei). It has terminations in one or more locations of gray matter in the *central nervous system* (CNS), where it synapses, the locations are not always well-known. A tract is made of several bundles of fibers, possibly not completely identified. Between the origin and the termination, the path may be short between near structures or long between brain segments, either crossing the midline or not.

Our description of tracts is in line with other studies such as the Foundational Model of Connectivity (FMC) developed by Swanson and Bota (2010) which is the basis of the representation of white matter tracts in the Terminologia Neuroanatomica (TNA, 2017). The TNA is a recent revision of the terminology on the CNS, the peripheral nervous system (PNS) and the sensory organs. These were abstracted from the Terminologia Anatomica (TA, 1998) and the Terminologia Histologica (TH, 2008) and were extensively updated by the Neuroanatomy Working Group of the Federative International Programme for Anatomical Terminology (FIPAT) of the International Federation of Anatomical Associations (IFAA), and was merged to form the TNA (ten Donkelaar et al., 2017; TNA, 2017), which currently stands alone. The FMC presentation of the connectome, with its three levels of connections—macroconnections (white matter tracts), mesoconnections (dealing with neuron types), and microconnections (dealing with individual neurons of a neuron type)—is a pertinent schema of representation, totally compatible with our approach. Most of the tract names in the TNA refer to macroconnections, because they reveal enough knowledge for naming a tract, whether the type of cells is reported or not. But there is no doubt that the documentation of mesoconnections in the future will help to differentiate new structures with consequent updates in the terminology.

Today, anatomical terminologies are challenged by the emergence of ontology and its formal approach. There was a time for the atlas of anatomy (and this approach is still valid), and there is a time for computer-based presentations. The basis of modern terminology for the biomedical sciences is the Basic Formal Ontology 2.0 (Smith et al., 2015), on which the FMA and our model are grounded. As seen in **Table 2** below, we adopt the taxonomy developed by the FMA (Rosse and Mejino, 2003). The rules governing the taxonomy and the partonomy have been made explicit and they are imperative. An expected benefit is to favor the exchange of data between computer applications. But the overall goal is a precise definition of anatomical entities. This will allow a better understanding among people living in distinct communities, with a specific background, while communicating in different languages. The past terminologies are the initial background for future developments, while modern terminologies are now ready to take the lead. This paper positions itself as a contribution to the advent of a modern terminology.

We have observed that anatomists may be reluctant to ontologies and related formal approaches.

“*Terminologies should not be developed by reference to a system of preferred terms, rather they should be developed in such a way that their individual nodes and relations amongst these nodes are modeled on an underlying formal ontology, where the linguistic content of these nodes will be filled in based on a system of terms and synonyms (from many different languages) that is associated with each node based on the intended ontological interpretation of that node.”*

(see Baud et al., 2007, where additional references may be found). Indeed, there is no opposition: ontologies are the necessary support of terminologies.

In a classic view of anatomical terminology, white matter is considered as a part of the brain segment where it appears. For example, in the Terminologia Anatomica (TA, 1998), the *posterior spinocerebellar tract* is described as a part of the *myelencephalon*. This entity is also present as a part of the *spinal cord*, but it is absent as part of the *cerebellum* where it terminates. Many tracts are mentioned several times in other segments, as if they are a part of it. The benefit of this approach was to document cross section views of brain segments and to make the path followed by the different tracks more explicit. This is an important aspect, but, hopefully, it is not lost in our new schema of presentation (see discussion below, see **Table 5**). The problem with such a presentation is that we depart from the rules of partonomy.

This is a classical error: it is well-known that the relation *CONTAINED_IN* differs from *PART_OF*. The *blood* is contained in the *vessels*, but not a part of them. The fact that a tract is crossing a brain segment does not makes this tract a part of it. If it did, because it crosses several segments, it would simultaneously be part of several brain segments. That is incompatible with the general statement of single inheritance in the partonomy: no entity can be a part of two entities. Therefore, it is formally incorrect to represent white matter tracts as part of the brain segments. The consequence is an ambiguous representation making it difficult to navigate in the knowledge base, as reported by several authors (Martin et al., 2001; Rosse, 2001; Rosse and Mejino, 2003; Bota and Swanson, 2008; Swanson and Bota, 2010; Swanson, 2014).

This classic view is widespread and shared by the TA (TA, 1998), the Foundational Model of Anatomy (FMA; Rosse and Mejino, 2003) and NeuroNames (Bowden and Martin, 1995; Martin et al., 2001; Bowden et al., 2012). Other initiatives generally claim to be compatible with any of these former studies. In the TA, the *pyramidal tract* is present as part of the *myelencephalon* and the *mesencephalon* with different codes. In the FMA, the *pyramidal tract* has no partonomic information so far. In NeuroNames, the *pyramidal tract* is part of the *medulla* or *myelencephalon*. Those statements are incomplete, because the pyramidal tract passes from the *cerebral cortex* to the *spinal cord*: only some segments of the pyramidal tract could be considered as part of the brain segments as stated above. But is it acceptable to state that *myelencephalic segment of pyramidal tract PART_OF myelencephalon* instead of *myelencephalic segment of pyramidal tract PART_OF pyramidal tract*? Certainly not. It is time to adjust our representation of tracts.

## New View on Tracts

On the contrary, as a general statement, the tracts are not a part of any segment, but should rather be considered in a separate section of the CNS as previously advocated in the Jenaer Nomina Anatomica (JNA, 1936), which contains the collection of all tracts and only those. This places a direct focus on the connections running through the white matter. The current number of tracts is about 100 items, but this number may grow in the coming years due to new discoveries.

What we propose is that tracts form directly part of the CNS (see Table 1). When concentrating all tracts in a single section, it is necessary to classify these tracts based on natural acceptable criteria. The origin of a tract is finally considered as the primary criterion, because it is always known and relatively well-localized to a single location, in contrast to terminations of tracts that are numerous and not always completely documented. On this basis, the tracts of the CNS are classified by their origin in the following 9 segments: *telencephalon (pallium), telencephalon (subpallium), hypothalamus, diencephalon, mesencephalon, cerebellum, rostral rhombencephalon, caudal rhombencephalon*, and *spinal cord*.

**Table 1:** Generic partonomy of tracts.

**Nervous system**	**Systema nervosum**
Central nervous system	Systema nervosum centrale
Tracts of central nervous system	Tractus systematis nervosi centralis
Tracts of origin	Tractus originis
Tracts of origin in segment 1	Tractus originis segmenti 1
Central roots	Radices centrales
Commissural tracts	Tractus commissurales
Intrinsic tracts	Tractus associationis; tractus proprii
Long tracts	Tractus longi
Ascending tracts	Tractus ascendentes
Descending tracts	Tractus descendentes
Tracts of origin in segment 2	Tractus originis segmenti 2
Mixed tracts	Tractus mixti

*The numbers represent specific segments of the CNS. Plural terms represent sets of entities and the hierarchical relation between two plural terms is subset_of interpreted as a specialization of part_of*.

In the TNA, a more natural hierarchical classification of brain structures is used for the prosencephalon (forebrain) as implemented in the revised version of the Terminologia Embryologica (TE2, 2017). The forebrain is subdivided into the caudal prosencephalon, giving rise to the diencephalon (pretectum, thalamus with epithalamus, prethalamus, and the prerubral or diencephalic tegmentum), and a rostral or secondary prosencephalon, giving rise to the hypothalamus and the entire telencephalon. The telencephalon is divided into the pallium and the subpallium (striatum, pallidum, basal forebrain, and preoptic area).

The diencephalon in its classic, columnar view (Herrick, 1910) was divided into four dorsoventrally arranged columns separated by ventricular sulci, i.e., the epithalamus, the dorsal thalamus, the ventral thalamus and the hypothalamus. It should be noted that NeuroNames and the FMC still follow this doctrine. Extensive embryological studies (see Puelles et al., 2013; ten Donkelaar et al., 2017, 2018) made it clear that the thalamic “columns” are derived from transversely oriented zones, the prosomeres which, from caudal to rostral, contain, in their alar domains, the pretectum (prosomere 1 or P1), the epithalamus and the thalamus (P2), and the prethalamus and the eminentia thalami (P3). The diencephalic basal plate contains the diencephalic part of the substantia nigra–VTA complex, the interstitial nucleus of Cajal, the nucleus of Darkschewitsch and the fields of Forel, collectively known as the prerubral or diencephalic tegmentum. The entire hypothalamus arises from the alar and basal components of the secondary prosencephalon. The preoptic region arises from the subpallium, but for practical reasons it is listed preceding the hypothalamus. The Kuhlenbeck (1967-1978) terms “interbrain” for diencephalon, and “afterbrain” for myelencephalon, as advocated in the FMC, are not widely recognized.

The term “pons,” as used in colloquial neuroanatomy for the rostral part of the hindbrain, is replaced by the term rostral rhombencephalon, keeping the term pons for the pons proper (the basilar part of the pons in most studies). For the caudal part of the hindbrain, the myelencephalon, the term caudal rhombencephalon is suggested.

Our choice of origin as the primary classification criterion is in accordance with the FMC. The first level or macroconnection is based on the region including the origin of a tract, whereas the second level, mesoconnection, is based on cell types. Therefore, our first criterion corresponds to the FMC first level.

A second criterion was necessary for the classification within a segment. The type of tract was selected for this purpose. The following subdivision was used (see Tables 1, 2):

1. *Central roots*, defined as the white matter tracts of the CNS that contribute (a) to cranial nerve roots within the brain stem, including the genu of the facial nerve, the decussation of the trochlear nerve, the spinal and mesencephalic tracts of the trigeminal nerve, and the solitary tract; and (b) the central projections of the dorsal roots of the spinal cord, forming the gracile and cuneate fasciculus. The optic tract may also be viewed as a central root.
2. *Intrinsic tracts*, restricted to a particular part of the brain, include: (a) *association tracts*, the association pathways of the telencephalon; (b) *intrinsic tracts* of other parts of the brain; and (c) the *intrinsic, propriospinal tracts* of the spinal cord.
3. *Commissural tracts*, connecting left and right parts of the brain or spinal cord segment, including the corpus callosum, the anterior, habenular, posterior and supraoptic commissures, and commissural connections in the spinal cord.
4. *Long tracts* connect various segments of the CNS and can be divided into (a) *ascending tracts*; (b) *cerebellar efferent tracts*; and (c) *descending tracts*, irrespective of whether they cross the midline or not.

**Table 2:** Taxonomy of tract.

Anatomical entity
Physical anatomical entity
Material anatomical entity
Anatomical structure
Postnatal anatomical structure
Cell part cluster
Cell part cluster of central nervous system
Tract of central nervous system
Tract of brain
Tract of origin in telencephalon
Central root of telencephalon
Commissural tract of origin in telencephalon
Association tract of origin in telencephalon
Descending tract of origin in telencephalon
Tract of origin in hypothalamus
Tract of origin in diencephalon
Central root of diencephalon
Commissural tract of origin in diencephalon
Association tract of origin in diencephalon
Long tract of origin in diencephalon
Ascending tract of origin in diencephalon
Descending tract of origin in diencephalon
Tract of origin in mesencephalon
Tract of origin in cerebellum
Tract of origin in rostral rhombencephalon
Tract of origin in caudal rhombencephalon
Tract of spinal cord
Tract of origin in spinal cord

*This is a partial expansion of the global taxonomy. All indentations in the hierarchy represent the ISA relation*.

In order to illustrate the use of the two criteria, three examples of partonomy are presented here:

1. **long tracts of origin in telencephalon** **>** **descending tracts of origin in telencephalon** **>** pyramidal tract > corticorubral fibers.
2. **tracts of origin in hypothalamus** **>** **efferent tracts of hypothalamus** **>** hypothalamohypophysial tracts > paraventriculohypophysial tract.
3. **long tracts of origin in spinal cord** **>** **ascending tracts of origin in spinal cord** **>** anterolateral tract > spinoreticular tract.

Once a tract has been classified according to its origin, it is possible to give more information on its path through different brain segments. To do that, it is possible in a partonomic view to present the path of this tract, decomposed in ipsilateral and contralateral segments and decussations as well. These parts of a track may be referenced in the partonomic presentation of the white matter of the brain segments.

An open question remains: how will this model evolve when new discoveries are made? In the domain of TNA, massive data are collected, and new investigation tools are available. There is no doubt that integration of new information will be necessary in the future. There are two open solutions. (1) the origin of tracts may be further defined, and the tract could be split up into subtracts corresponding to detailed origins; (2) different types of cells (mesoconnection) at the origin of a tract may be used to differentiate the tract into several tracts. This paper does not answer this question, however, the new schema formally improves the approach relative to the traditional approach and is consequently better adapted to the evolution of knowledge.

## Integration in Taxonomy

Today, there is a single valid taxonomy for the domain of human anatomy, provided by the FMA (Rosse and Mejino, 2003). The emergence of an alternate taxonomy, although theoretically possible, has little chance, because such an enterprise has a workload of several years, currently not available, with no promise of an improved solution. The scientific community must therefore concentrate on the existing FMA taxonomy, and create collaborations to accommodate further improvements. And indeed, the actual FMA taxonomy is of significant value.

The role of the taxonomy is important in a modern ontology. The traditional presentation by the anatomists is the atlas of anatomy, inspired by the partonomic hierarchy. The atlas paradigm is convenient for the inventory of the relevant anatomical entities. Because past terminologies essentially were inventories of the domain, this approach was convenient. But today, a modern terminology must include a classification schema. The taxonomy, based on the genus and differentia principle of Aristotle, is the answer of choice to this need. Consequently, any anatomical entity is positioned in both hierarchies: partonomy and taxonomy. For example: *humerus isa long bone* and *humerus partof free upper limb*.

Apparently, the FMA taxonomy was not recently updated to correspond with the many new developments in neuroanatomy. Therefore, a revision of the neuroanatomical subdivision, based largely on NeuroNames, would be welcome. The present study offers a contribution to such a revision. Currently, the white matter that is essentially made of axons is classified (see Table 2) as a *cell part cluster of central nervous system* and its main child is *tract of central nervous system* that contains the majority of tracts. There are roughly 100 tracts classified into two groups: *tract of brain* and *tract of spinal cord*. Because this figure may be considerably augmented in the future, such a long flat list is no longer acceptable: further subdivisions must be created. Here, the above criterion may be inserted.

## Overview of The Tracts

Making an exhaustive inventory of tracts is not the goal of the terminology of today, when our knowledge of the CNS must be permanently updated by new discoveries. Therefore, the presentation of tracts is made of open lists. There are basically two conditions to fulfill before a new tract may enter the tables: (1) it should be recognized as important; and (2) it should have reached a significant level of agreement in the domain. These criteria are obviously subjective: it is the responsibility of the authors of the terminology to decide what is present or not. These two conditions should obviously be updated permanently, making the terminology subject to continual changes.

As seen above, tracts are grouped into 9 segments. In this paper, only tracts originating in the diencephalon are presented. An extended version of this paper, including all segments, is available by the authors.

The tables related to each segment are exclusively partonomic: each indentation means a *part_of* relation between the father entity and the indented entity. Plural terms refer to a set of entities. The link from a plural to a plural term must be understood as a *subset_of* and from a plural to a singular term as a *member_of*, both being a specialization of a *part_of*.

## The Tracts of Origin in The Diencephalon

The *tracts of origin in diencephalon* (*tractus originis diencephali*) include a central root, commissural, intrinsic and long tracts, ascending as well as descending (Table 3). The unique *central root* in the diencephalon is the *optic tract* (*tractus opticus*) with three distinct parts, the *lateral root* (*radix lateralis*), the *medial root* (*radix medialis*), and the *retinohypothalamic tract* (*tractus hypothalamicus*). Since the optic tract, arising from the retinal ganglion cells, mainly terminates in the lateral geniculate body, it is defined as a diencephalic central root. There are two crossing bundles of fibers, the *habenular commissure* (*commissura habenulae*), connecting the epithalami, and the *posterior commissure* (*commissura posterior*), a commissure of the pretectum. Intrinsic to the diencephalon are four tracts, the *external medullary lamina* (*lamina medullaris lateralis)*, the *internal medullary lamina* (*lamina medullaris medialis*), *intrathalamic fibers* (*fibrae intrathalamicae*), and *periventricular fibers* (*fibrae periventriculares*).

**Table 3:** Tracts of origin in the diencephalon.

**Tracts of origin in diencephalon**	**Tractus originis diencephali**
**Central roots of diencephalon**	**Radices centrales diencephali**
Optic tract	Tractus opticus
Lateral root	Radix lateralis
Medial root	Radix medialis
Retinohypothalamic tract	Tractus retinohypothalamicus
**Commissural tracts of origin in diencephalon**	**Tractus commissurales originis diencephali**
Habenular commissure	Commissura habenulae
Posterior commissure	Commissura posterior
**Intrinsic tracts of origin in diencephalon**	**Tractus proprii originis diencephali**
External medullary lamina	Lamina medullaris lateralis
Internal medullary lamina	Lamina medullaris medialis
Intrathalamic fibers	Fibrae intrathalamicae
Periventricular fibers	Fibrae periventriculares
**Long tracts of origin in diencephalon**	**Tractus longi originis diencephali**
**Ascending tracts of origin in diencephalon**	**Tractus ascendentes originis diencephali**
Thalamostriatal fibers	Fibrae thalamostriatales
Subthalamopallidal fibers	Fibrae subthalamopallidales
Thalamic radiations	Radiationes thalamicae
Anterior thalamic radiation; anterior radiation	Radiatio anterior thalami
Thalamofrontal fibers	Fibrae thalamofrontales
Central thalamic radiation; central radiation	Radiatio centralis thalami
Thalamoparietal fibers	Fibrae thalamoparietales
Inferior thalamic radiation; inferior radiation	Radiatio inferior thalami
Thalamotemporal fibers	Fibrae thalamotemporales
Posterior thalamic radiation; posterior radiation	Radiatio posterior thalami
Acoustic radiation	Radiatio acustica
Optic radiation; geniculocalcarine fibers	Radiatio optica; fibrae geniculocalcarinae
Anterior bundle	Fasciculus anterior
Central bundle	Fasciculus centralis
Dorsal bundle	Fasciculus dorsalis
Ascending peduncular fasciculus	Fasciculus peduncularis ascendens
**Descending tracts of origin in diencephalon**	**Tractus descendentes originis diencephali**
Habenulointerpeduncular tract; fasciculus retroflexus	Tractus habenulointerpeduncularis; fasciculus retroflexus
Medial tegmental tract	Tractus tegmentalis medialis
Pretectoolivary tract	Tractus pretectoolivaris
Prerubroolivary tract	Tractus prerubroolivaris
Descending medial longitudinal fasciculus	Fasciculus longitudinalis medialis descendens
Interstitiospinal tract	Tractus interstitiospinalis

There are three groups of *ascending fibers*: the *thalamostriatal fibers* (*fibrae thalamostriatales*), the *subthalamopallidal fibers* (*fibrae subthalamopallidales*), and the extensive connections of the thalamus to the cerebrum, known by the generic name *thalamic radiations* (*radiationes thalamicae*). These radiations, anterior, central, inferior and posterior (see Table 3) reach each lobe of the cerebrum. The *acoustic radiation* (*radiatio acustica*) projects to the temporal lobe, the *optic radiation* (*radiatio optica*) to the occipital lobe, whereas the *ascending peduncular fasciculus* (*fasciculus peduncularis ascendens*) connects the thalamus to the claustrum. This mixed tract (see Table 4) also contains fibers from the claustrum to the thalamus (the *descending peduncular fasciculus*).

**Table 4:** Mixed tracts.

**Mixed tracts of central nervous system**	**Tractus mixti systematis nervosi centralis**
Peduncular fasciculus	Fasciculus peduncularis
>Ascending peduncular fasciculus	>Fasciculus peduncularis ascendens
>Descending peduncular fasciculus	>Fasciculus peduncularis descendens
Ansa peduncularis	Ansa peduncularis
>Thalamotemporal fibers	>Fibrae thalamotemporales
>Corticothalamic fibers	>Fibrae corticothalamicae
>Ventral amygdalofugal tract	>Tractus amygdalofugalis ventralis
Medial fasciculus of prosencephalon	Fasciculus prosencephali medialis
>Ascending medial fasciculus of prosencephalon	>Fasciculus prosencephali medialis ascendens
>Descending medial fasciculus of prosencephalon	>Fasciculus prosencephali medialis descendens
Posterior longitudinal fasciculus; dorsal longitudinal fasciculus	Fasciculus longitudinalis posterior; fasciculus longitudinalis dorsalis
>Ascending posterior longitudinal fasciculus	> Fasciculus longitudinalis posterior ascendens
>Descending posterior longitudinal fasciculus; descending dorsal longitudinal fasciculus	>Fasciculus longitudinalis posterior descendens; fasciculus longitudinalis dorsalis descendens
Medial longitudinal fasciculus	Fasciculus longitudinalis medialis
>Ascending medial longitudinal fasciculus	>Fasciculus longitudinalis medialis ascendens
>Descending medial longitudinal fasciculus	>Fasciculus longitudinalis medialis descendens

*The sign “>” means a reference to another table of which the tract is a part. The present list is not exhaustive and limited to the main mixed tracts*.

The descending fibers arising in the diencephalon include: (1) the *habenulointerpeduncular tract* or *fasciculus retroflexus* (*tractus habenulointerpeduncularis* or *fasciculus retroflexus*) from the habenular nuclei to the mesencephalic interpeduncular nucleus; (2) the *medial tegmental tract* (*tractus tegmentalis medialis*), arising in the pretectum and the prerubral tegmentum, projecting to the inferior olivary complex, and composed of the *pretectoolivary tract* (*tractus pretectoolivaris*) from the pretectum, and the *prerubroolivary tract* (*tractus prerubrolivaris*) from the elliptic nucleus (the nucleus of Darkschewitsch) in the prerubral tegmentum; (3) the *descending medial longitudinal fasciculus* (*fasciculus longitudinalis medialis descendens*), containing the *interstitiospinal tract* (*tractus interstitiospinalis*), arising in the interstitial nucleus of Cajal.

## Mixed Tracts

*Mixed tracts* have ascending and descending components, using the same tract, exemplified by the *medial fasciculus of the prosencephalon*, also known as the *medial forebrain bundle*. See Table 4 for a list of mixed tracts.

## Rostrocaudal Sequencing

A general rule has been adopted for the sequence of entities, when there are several children from a father entity. In general, in a partonomic hierarchy, the order of children is open. It was decided to always sequence the tracts from the most rostral to the most caudal, when this sequence is significant. In other situations, like the *spinal laminae*, the sequence is determined by the current usage, here from posterior to anterior position.

## Brain Segment White Matter

It was also necessary to make explicit the links between the white matter representation in a segment and the tracts originating, terminating and traversing this segment. Because the representation is done by a partonomy, and the tracts are no more a part of the segment, we used a convention, named a *reference*. In a brain segment, we were concerned with tracts of several origins. We had to point to all of them in a specific order, proper to the segment. A reference is a pointer to an anatomical entity located elsewhere in the partonomic hierarchy. It is here represented by the sign “>.”

Typically, in the representation of the white matter of the cerebellum, we would point to the tracts running in the *brachium conjunctivum* using references. The long tracts using this pathway to enter or exit the cerebellum are not part of it and can only be revealed by a reference. See Table 5 about the white matter of the cerebellum.

**Table 5:** White matter of cerebellum.

**White matter of cerebellum**	**Substantia alba cerebelli**
Arbor vitae	Arbor vitae
Medullary body of cerebellum	Corpus medullare cerebelli
Medullary lamella of cerebellum	Lamella medullaris cerebelli
Cerebellar cortical afferent fibers	Neurofibrae afferentes corticis cerebelli
Mossy fibers	Neurofibrae muscosae
Climbing fibers	Neurofibrae ascendentes
Multilayered fibers; monoaminergic fibers	Neurofibrae multistratificatae
Cerebellar cortical efferent fibers	Neurofibrae efferentes corticis cerebelli
Axons of Purkinje cell	Axones neuri purkinjensis
>Commissural tracts of origin in the cerebellum	>Tractus commissurales originis cerebelli
>Cerebellar commissure	>Commissura cerebelli
Cerebellar peduncles	Pedunculi cerebellares
Superior cerebellar peduncle	Pedunculus cerebellaris superior
Brachium conjunctivum	Brachium conjunctivum
Ascending branch	Ramus ascendens
>Cerebellorubral tract	>Tractus cerebellorubrale
>Cerebellothalamic tract	>Tractus cerebellothalamicum
Descending branch	Ramus descendens
>Uncinate fasciculus of cerebellum	> Fasciculus uncinatus cerebelli
>Fastigiobulbar fibers	> Fibrae fastigiobulbares
>Fastigiospinal tract	> Tractus fastigiospinalis
>Interpositospinal tract	> Tractus interpositospinalis
>Anterior spinocerebellar tract; ventral spinocerebellar tract	>Tractus spinocerebellaris anterior; tractus spinocerebellaris ventralis
Middle cerebellar peduncle	Pedunculus cerebellaris medius
>Pontocerebellar fibers	>Fibrae pontocerebellares
Inferior cerebellar peduncle	Pedunculus cerebellaris inferior
Restiform body	Corpus restiforme
>Cerebelloolivary fibers; nucleoolivary fibers	>Fibrae cerebelloolivares; fibrae nucleoolivares
>Posterior spinocerebellar tract; dorsal spinocerebellar tract	>Tractus spinocerebellaris posterior; tractus spinocerebellaris dorsalis
>Cuneocerebellar fibers	>Fibrae cuneocerebellares
>Trigeminocerebellar fibers	>Fibrae trigeminocerebellares
>Olivocerebellar tract	>Tractus olivocerebellaris
>Nucleocerebellar fibers	>Fibrae nucleocerebellares
>Raphecerebellar fibers	>Fibrae raphecerebellares
Juxtarestiform body	Corpus juxtarestiforme
>Vestibulocerebellar fibers	>fibrae vestibulocerebellares
>Cerebellovestibular fibers	>Fibrae cerebellovestibulares
Peduncle of flocculus	Pedunculus flocculi

*The sign “>” means a reference to another table of which the tract is a part*.

## Conclusion

The new schema for white matter gives a direct focus on the several tracts of the central nervous system. It brings a formally correct representation that is necessary for further developments of a highly developing domain.

The need to improve the formal aspect of a modern terminology has been underlined. The dual approach with two facets–the partonomy and the taxonomy–becomes evidence. Multiple computer applications are developed today, and they will exchange their data only on the condition that they have a common background: the taxonomy plays this role. The terminological aspect remains a predominant source of problems and a universal consensus has not yet been reached. However, sound principles as exemplified in this paper contribute to the solutions of tomorrow.

## Author Contributions

All authors listed have made a substantial, direct and intellectual contribution to the work, and approved it for publication.

### Conflict of Interest Statement

The authors declare that the research was conducted in the absence of any commercial or financial relationships that could be construed as a potential conflict of interest. The reviewer RR and handling Editor declared their shared affiliation at the time of review.

## References

[B1] Baud R. Ceusters W. Ruch P. Rassinoux A. M. Lovis C. Geissbühler A. (2007). Reconciliation of ontology and terminology to cope with linguistics. Stud. Health Technol. Inform. 29(Pt. 1):796–801. 17911826

[B2] Bota M. Swanson L. W. (2008). BAMS neuroanatomical ontology: design and implementation. Front. Neuroinform. 2:8. 10.3389/neuro.11.002.2008 18974794 PMC2525975

[B3] Bowden D. M. Martin R. F. (1995). NeuroNames brain hierarchy. Neuroimage 2, 63–83. 10.1006/nimg.1995.1009 9410576

[B4] Bowden D. M. Song E. Kosheleva J. Dubach M. F. (2012). neuronames: an ontology for the braininfo portal to neuroscience on the web. Neuroinformatics 10, 97–114. 10.1007/s12021-011-9128-8 21789500 PMC3247656

[B5] Herrick C. J. (1910). The morphology of the forebrain in amphibia and reptilia. J. Comp. Neurol. 20, 413–547 10.1002/cne.920200502

[B6] JNA Jenaer Nomina Anatomica. (1936). Approved June 1935 by the Anatomische Gesellschaft in Jena, Published Early 1936 by H.Stieve. Jena: Fischer

[B7] Kuhlenbeck H (1967-1978). The Central Nervous System of Vertebrates, Vol 5 Basel: Karger.

[B8] Martin R. F. Mejino J. L. V. Jr Bowden D. M. Brinkley J. F. Rosse C. (2001). Foundational model of neuroanatomy: implications for the human brain project. Proc. AMIA Symp. 2001, 438–442. PMC2243655 11825226

[B9] Puelles L. Harrison M. Paxinos G. Watson C. (2013). A developmental ontology for the mammalian brain based on the prosomeric model. Trends Neurosci. 36, 570–578. 10.1016/j.tins.2013.06.004 23871546

[B10] Rosse C. (2001). Terminologia Anatomica: considered from the perspective of next-generation knowledge sources. Clin. Anat. 14, 120–133. 10.1002/1098-2353(200103)14:2<120::AID-CA1020>3.0.CO;2-V 11241747

[B11] Rosse C. Mejino J. L. V Jr. (2003). A reference ontology for biomedical informatics: the foundational model of anatomy. J. Biomed. Inform. 36, 478–500. 10.1016/j.jbi.2003.11.007 14759820

[B12] Smith B. Almeida M. Bona J. Brochhausen M. Ceusters W. Courtot M. (2015). BFO 2.0 Specification and User's Guide. Available online at: https://raw.githubusercontent.com/BFO-ontology/BFO/v2.0/BFO2-Reference.docx

[B13] Swanson L. W. (2014). Neuroanatomical Terminology. A Lexicon of Classical Origins and Historical Foundations. New York, NY: Oxford University Press.

[B14] Swanson L. W. Bota M. (2010). Foundational model of structural connectivity in the nervous system with a schema for wiring diagrams, connectome, and basic plan architecture. Proc. Natl. Acad. Sci. U. S. A. 107, 20610–20617. 10.1073/pnas.1015128107 21078980 PMC2996420

[B15] TA (1998) Terminologia Anatomica. Federative Committee on Anatomical Terminology. Stuttgart: Thieme.

[B16] TE2 (2017) Terminologia Embryologica. FIPAT.Library.dal.ca. Federative International Programme for Anatomical Terminology.

[B17] ten Donkelaar H. J. Broman J. Neumann P. E. Puelles L. Riva A. Tubbs R. S.. (2017). Towards a terminologia neuroanatomica. Clin. Anat. 30, 145–155. 10.1002/ca.22809 27910135

[B18] ten Donkelaar H. J. Kachlík D. Tubbs S. T. (2018). An Illustrated Terminologia Neuroanatomica: A Concise Encyclopedia of Human Neuroanatomy. Heidelberg: Springer.

[B19] TH (2008) Terminologia Histologica. Federative Committee on Anatomical Terminology. Philadelphia PA: Lippincott, Williams & Wilkins.

[B20] TNA (2017) Terminologia Neuroanatomica. FIPAT.Library.dal.ca. Federative International Programme for Anatomical Terminology.

